# Validation of Machine Learning-Aided and Power Line Communication-Based Cable Monitoring Using Measurement Data

**DOI:** 10.3390/s24020335

**Published:** 2024-01-05

**Authors:** Yinjia Huo, Kevin Wang, Lutz Lampe, Victor C.M. Leung

**Affiliations:** 1 Department of Electrical and Computer Engineering, The University of British Columbia, Vancouver, BC V6T 1Z4, Canada; yortka@ece.ubc.ca (Y.H.); kevin.wang@tum.de (K.W.);; 2 Electrical Engineering and Information Technology, Technical University of Munich, 80333 Munich, Germany; 3 College of Computer Science and Software Engineering, Shenzhen University, Shenzhen 518060, China

**Keywords:** power line communications (PLC), cable monitoring, machine learning (ML), unsupervised learning, smart grid, channel frequency response, principal component analysis (PCA), clustering

## Abstract

The implementation of power line communications (PLC) in smart electricity grids provides us with exciting opportunities for real-time cable monitoring. In particular, effective fault classification and estimation methods employing machine learning (ML) models have been proposed in the recent past. Often, the research works presenting PLC for ML-aided cable diagnostics are based on the study of synthetically generated channel data. In this work, we validate ML-aided diagnostics by integrating measured channels. Specifically, we consider the concatenation of clustering as a data pre-processing procedure and principal component analysis (PCA)-based dimension reduction for cable anomaly detection. Clustering and PCA are trained with measurement data when the PLC network is working under healthy conditions. A possible cable anomaly is then identified from the analysis of the PCA reconstruction error for a test sample. For the numerical evaluation of our scheme, we apply an experimental setup in which we introduce degradations to power cables. Our results show that the proposed anomaly detector is able to identify a cable degradation with high detection accuracy and low false alarm rate.

## 1. Introduction

### 1.1. Background and Motivation

Asset monitoring is an integral part of the modern smart grid [1]. Power cables are key grid assets, whose failure can have serious consequences for service provision, operational safety, and economics of operation. In light of this, preventive diagnostics relying on real-time monitoring of cable health conditions ([2] Ch. 6) is imperative.

The implementation of power line communication (PLC) modems in smart electricity grids provides us with exciting opportunities for real-time cable monitoring on live wires [3]. PLC modems use the existing power line infrastructure as the communication medium, making PLC an appealing solution to communication requirements in smart grids [3]. Changes in the electric properties of the grid, including those caused by cable degradation, also manifest themselves as changes in the communication medium used by PLC, thus affecting the PLC channel [4]. Through probing the PLC medium and analyzing the received communication signal, we are able to obtain real-time information about cable health conditions [5,6,7,8].

Many of the existing works using PLC for cable diagnostics are based on the study of synthetically generated channels, e.g., [5,6,7,8,9]. Synthetic channel generation can be either top-down or bottom-up. The top-down approach uses the statistical properties of a handful of PLC channel measurements for generation [10]. Specifically, the statistical properties of the generated synthetic channels are matched to those of measured PLC channels. However, this approach does not account well for the topology and the physical properties of the particular PLC network that we want to diagnose. On the other hand, the bottom-up approach uses transmission line theory to generate the synthetic channels for specific topologies and physical properties of the considered PLC network [11,12]. However, the bottom-up approach necessitates the use of idealized assumptions, and thus it cannot account (well) for all artefacts of practical channels. For example, cable joints and connecting pieces are often ignored, and assumptions about insulation parameters and homogeneity of insulation are only approximations for the scenarios found in practical settings. For these reasons, synthetically generated channels are not ideal matches for real PLC channels [13]. Therefore, the training and testing of learned PLC diagnostic methods considering actual measured channels is crucial for the validation of this line of work.

### 1.2. Challenges and Contributions

In this work, we use measured PLC channels to develop our cable diagnostics scheme. One of the challenges for developing a cable diagnostics scheme using measured channels is that it is costly and often infeasible to obtain a sufficient number of measurements with degraded cables. Therefore, in this work, we use an unsupervised machine learning (ML) method, which is solely developed based on the measurements in healthy conditions. This circumvents the use of measurements with degraded cables. In particular, we propose cable anomaly detection through the application of principal component analysis (PCA), with PLC channel frequency response (CFR) vectors as the input. Cable anomalies are indicative of incipient and already developed cable faults and thus support taking corrective or preventative measures to avoid a potential cable fault event.

To illustrate and validate the capabilities of ML-aided PLC cable monitoring, we train the PCA model using the CFR measurements of the overall network working under healthy conditions. More specifically, to obtain sufficiently many realistic PLC channel realizations for training, we apply a hybrid measurement–synthetic approach whereby measurement data are amended by synthetically generated load terminations that account for the random nature of the grid network impedance. Then, we test the trained model on CFR measurements of the PLC network, where anomalies are detected by considering the reconstruction error using the PCA representation of the measured data. To obtain the quantitative evaluation of the effectiveness of our developed cable anomaly detection scheme, we manually introduce degradations to the healthy cable, which are proxies for actual physical damages experienced by cables in a real-world scenario. As a proof of concept, the results in this paper are obtained using low-voltage power extension cables for measurements in a laboratory setting. While this setup is different from actual low- or medium-voltage power cables installed in distribution grids, we argue that the underlying PLC signal propagation principles apply equally and therefore we are able to provide meaningful insights with the used setup.

PCA-based anomaly detection is aided by data clustering as a pre-processing procedure. This is motivated by the fact that variations unrelated to anomalies are present in CFR features. Clusters are identified from the healthy training data and a PCA-based anomaly detector is developed for each cluster. This permits the anomaly detector to focus on the internal structure of the associated data cluster rather than accounting for the inter-cluster variance of the data. Performance of the proposed method is evaluated based on the trade-off between detection accuracy (DA) for the degraded cases and the false alarm rate (FA) for the healthy cases. Performance results suggest that the combination of clustering and PCA is highly effective for cable anomaly detection.

We briefly summarize the contributions of this work. First, we develop and evaluate our method using real measurements instead of synthetic data, as has often been performed in the literature. Second, we use an unsupervised ML approach, which alleviates the need for measurements for the faulty case during training. Third, we propose clustering as a data pre-processing procedure that significantly boosts performance. Finally, through numerical evaluations, our proposed cable anomaly detection scheme is shown to be effective, which is an important step towards validating the practical use of ML-aided PLC cable monitoring.

### 1.3. Related Work

Before proceeding with the details of the proposed method, we summarize relevant related work, starting with cable diagnostics schemes in the broad sense that do not necessarily employ PLC. A fairly recent example is [14], where relatively inexpensive sensors are installed across the grid as fault indicators. However, this approach only detects faults while our designed solution is able to respond to cable anomalies in general, including cable degradations such as those arising from, for example, water-treeing. Reference [15] uses the sampled voltage and current phasors at the main frequency to detect an anomaly as well as locate the phase of a three-phase power transmission system at which the anomaly occurs. Phasor measurement units (PMUs) are applied for fault detection in [16,17]. While [16] uses threshold-based event detection and supervised learning for classification, reference [17] develops distance-based outlier detection. The work in [18] uses a normalized traveling-wave and threshold method to identify a fault. Then, it aligns the measurement with a reference measurement to localize the fault. If measurements and indicators as considered in [15,16,17,18] are available, then they could be combined with the PLC-assisted anomaly detection scheme developed in this work to improve the quality of cable monitoring.

Various data-driven techniques have been applied for fault detection in smart grids, in particular different forms of neural network structures, e.g., [19,20,21,22]. However, these solutions are for fault detection, while our designed scheme is intended for cable anomalies in the broad sense. In particular, the identification of cable degradations enables us to also achieve fault prevention.

Furthermore, several cable diagnostics solutions based on the analysis of PLC signals have been developed in previous works such as [5,6,7,8,9,23,24,25,26,27,28,29,30,31,32]. References [23,24,30,31,32] use PLC signals to carry out time domain reflectometry analysis for fault detection and localization. In particular, in [23], a prototype hardware system is constructed and on-line results are presented, while in [24], the designed solution is applied to a line that is part of the Greek rural distribution system, and its validity is tested. In [30,31,32], test results based on laboratory experimentation are presented. However, the reflectometry technique only deals with concentrated cable anomalies while our solution is also sensitive to degradations that extend over a certain length. In [25], the PLC signal strength is found to be an indicator of abnormal behavior of smart meters in an experimental study. However, cable anomalies do not always cause a drop in PLC signal strength. Some of the solutions presented in the literature [5,27,28] compare the PLC signal to be analyzed to a reference signal representing the healthy cable state. However, since grid conditions are constantly changing due to varying loads and switching operations, it is difficult to infer whether differences between PLC signal samples are due to an anomaly or these operational changes. Some other works such as [8,29,33] use ML methods to intelligently detect and assess cable health. However, they typically apply supervised learning by training the ML models with PLC signal samples obtained under both healthy and degraded conditions. In contrast, our work makes use of unsupervised learning, as it does not necessitate the inclusion of PLC signal samples under the anomalous condition, which are difficult and costly to obtain. Moreover, many works, e.g., [5,8,26,27,28,29,34,35], are based on the study of synthetically generated PLC channel realizations. This necessarily entails idealized assumptions such as, for example, homogeneity of insulation material along the entire cable.

The concept of anomaly detection using unsupervised learning and PLC signals as input is not new. Our previous work [36] applied this approach, albeit using time-series prediction for indicating an anomaly. More specifically, PLC modems continuously report signal-to-noise ratio (SNR) measurements that are processed in a trained predictor. In contrast to this, the PCA-based anomaly detector developed in this work processes individual channel measurements. This renders it applicable also in scenarios where time-series processing is impossible due to the absence of corresponding data or undesirable due to the required continuous processing. Otherwise, one could consider the methods proposed in this work and in [36] as complementary, and they could be combined as an ensemble method. Also, the scheme presented in [37] works on unsupervised learning based on SNR measurements. It uses feature dimension reduction, clustering, and classification to observe transitions in data-to-cluster mappings, which indicate changes in the monitored link and thus changes in the grid. The work presented in this paper also uses clustering techniques but as a data pre-processing step so as to train and apply separate anomaly detectors for each data cluster. As illustrated later in Section 4, data clusters are found to correspond to different load conditions for healthy cables, i.e., clustering identifies non-anomalous changes in the grid, and thus it is challenging to base anomaly detection on clustering only.

### 1.4. Organization of the Paper

The remainder of this paper is organized as follows. We provide the technical background for the measurement procedures including applied cable degradations in Section 2. Then, we describe clustering, PCA, and their implementation for our unsupervised cable anomaly detection scheme in Section 3. Results for the measurements, clustering, and the PCA-based anomaly detection are presented and discussed in Section 4. Finally, the conclusions are drawn in Section 5.

## 2. Experimental Setup

**Figure 1:**
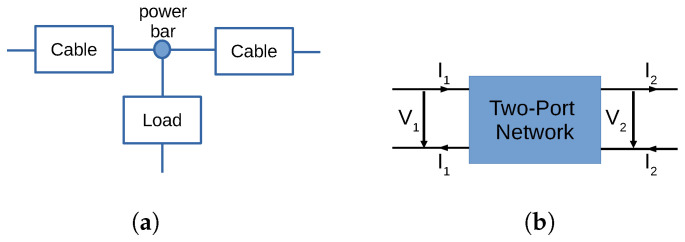
T−network arrangement and two−port model used for experiments and analysis. (**a**) Schematics of the T−Network arrangement used for measurements. (**b**) A two−port network with signal voltages and currents.

We consider a T-network arrangement in our experimental setup as shown in Figure 1a. Two of the three branches of the T-network are realized by two 30 m 12-gauge 15 A power cables, which are connected through a standard 6-outlet power bar with surge protection. The power bar is plugged into the wall socket. Additional loads are connected to the power bar as well, forming the third branch of the T-network.

We consider this setup as a mock-up of an actual grid environment consisting of different electric components, including segments of sample medium-voltage cables, cable splices, various loads, etc., which permits us to conduct experiments in a controlled laboratory environment. While further measurements on (replicas of) actual grid deployments are desirable as a next step, we believe that this is a meaningful configuration for the validation of ML-aided PLC cable monitoring.

The measurements are performed using a vector network analyzer (VNA), representing the system between the measurement points as a two-port network. Since the VNA measures S-parameters and we use ABCD parameters for the generation of synthetic training data, we first briefly review some relevant technical background in Section 2.1. Then, we provide details on the measurement procedures in Section 2.2 and Section 2.3.

### 2.1. Two-Port Networks

The electrical behavior of a two-port network that is composed of linear electrical components operating under steady-state electrical signal stimuli can be described using its four scattering parameters (S-parameters) $S_{11},S_{12},S_{21}$ and $S_{22}$. The S-parameters relate the incident power waves $a_{1}$ and $a_{2}$ propagated towards the two-port network at Port 1 and Port 2, respectively, and the reflected power waves $b_{1}$ and $b_{2}$ propagated outward from the two-port network at Ports 1 and 2, respectively, as

(1) $$\left[\begin{array}{c} b_{1}\\ b_{2} \end{array}\right]=\left[\begin{array}{cc} S_{11} & S_{12}\\ S_{21} & S_{22} \end{array}\right]\left[\begin{array}{c} a_{1}\\ a_{2} \end{array}\right].$$

While the S-parameters can be obtained through measurements with a VNA, it is more convenient to consider the ABCD parameters of the two-port network to analyze PLC signal propagation. The ABCD parameters relate the voltages and currents as defined in Figure 1b via

(2) $$\left[\begin{array}{c} V_{1}\\ I_{1} \end{array}\right]=\left[\begin{array}{cc} A & B\\ C & D \end{array}\right]\left[\begin{array}{c} V_{2}\\ I_{2} \end{array}\right].$$

Given the S-parameters and a reference impedance $Z_{0}$ used by the VNA during the measurements, the ABCD parameters can be obtained from [38]

(3) $$\left[\begin{array}{cc} A & B\\ C & D \end{array}\right]=\left[\begin{array}{cc} \frac{\left(1+S_{11}\right)\left(1-S_{22}\right)+S_{12}S_{21}}{2S_{21}} & Z_{0}\frac{\left(1+S_{11}\right)\left(1+S_{22}\right)-S_{12}S_{21}}{2S_{21}}\\ \frac{1}{Z_{0}}\frac{\left(1-S_{11}\right)\left(1-S_{22}\right)-S_{12}S_{21}}{2S_{21}} & \frac{\left(1-S_{11}\right)\left(1+S_{22}\right)+S_{12}S_{21}}{2S_{21}} \end{array}\right].$$

### 2.2. Calibration

**Figure 2:**
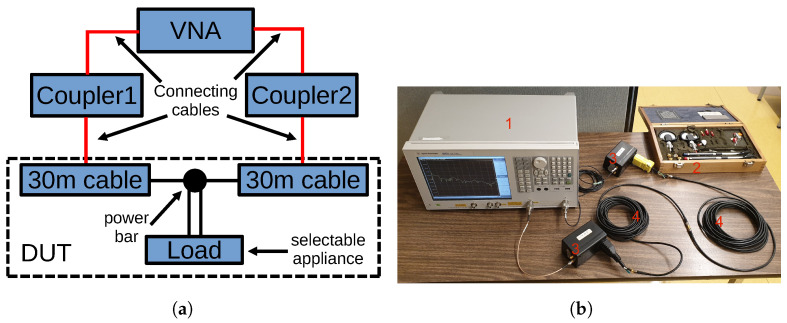
Illustration of the measurement and calibration setup. (**a**) Schematics for DUT and ancillary components. (**b**) Photo of the calibration setup (through measurement). Labelled items are 1—VNA, 2—calibration kit, 3—couplers, 4—connection cables.

Calibration is a key step in our measurement setup. As shown in Figure 2a, the VNA is connected to the actual device under test (DUT) through PLC couplers and connection cables. The calibration de-embeds those ancillary components so as to measure the S-parameters of the DUT network. We perform calibration using a commercial calibration kit and applying the usual short, open, load and through measurements. Figure 2b shows the setup for the through measurement. Once the calibration is completed, the VNA measurements give us the S-parameters of the DUT, where the two 30 m cables are connected to a power bar with several appliances, as explained in Section 2.3 below. From these, the ABCD parameters can be obtained as per (3).

### 2.3. Measurements

The measurements are conducted in two states: a non-energized and an energized setup. As mentioned, PLC couplers are used at each port of the VNA to protect it in the case of measurements on the energized setup. The VNA is an E5061B from Agilent Technologies with the frequency range of 100 kHz to 3 GHz, an adjustable resolution, and reference impedance $Z_{0}=50$ Ω. Furthermore, coaxial cables are used between VNA and coupler and between coupler and DUT to reduce the mechanical stress on the VNA ports and to minimize the effects of changes to the physical layout. Due to the calibration procedure mentioned in the previous step, the influence of those coaxial cables as well as the influence of the couplers to the final measurement result are removed.

**Figure 3:**
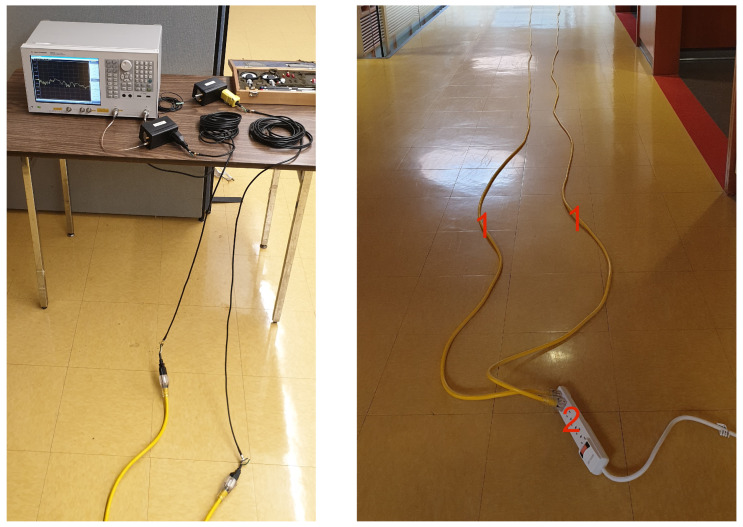
Photos of the measurement setup with the layout of the 30 m cables. Labelled items are 1—low-voltage power extension cables, 2—power bar.

**Figure 4:**
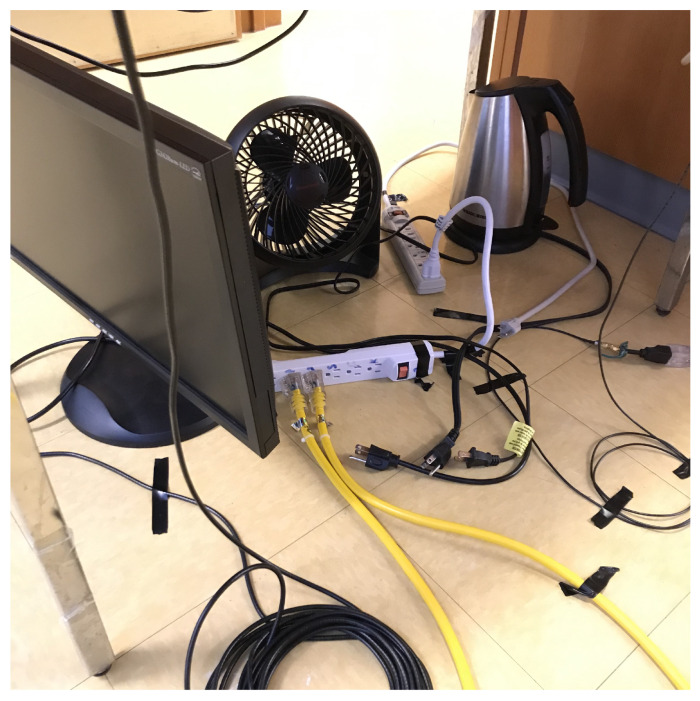
Photo of the three loads unplugged from the power bar.

Figure 3 shows the basic layout of the measurement setup. Two 30 m power cables are connected to the two ports of the VNA on one end and to the power bar on the other end. The cables are laid out in straight lines. The power bar is either connected to a power outlet in the wall (energized setup) or with its plug left unconnected (non-energized setup). Three different loads, a fan, a kettle and a computer monitor are connected to the power bar in all eight possible configurations, i.e., from none to all three loads being plugged in, as enumerated in Table 1. Figure 4 shows the case of all three loads unplugged from the power bar. The fan has a power consumption of 48 W, the kettle consumes 1500 W, and the computer monitor has a power consumption of approximately 60 W.

### 2.4. Manually Applied Degradations

For the numerical evaluation of the cable anomaly detection scheme proposed in Section 3, we manually introduce degradations to the healthy cable in seven stages. These include insulation removal, strand removal, and shorting of conductors as explained in detail in the following.

**Figure 5:**
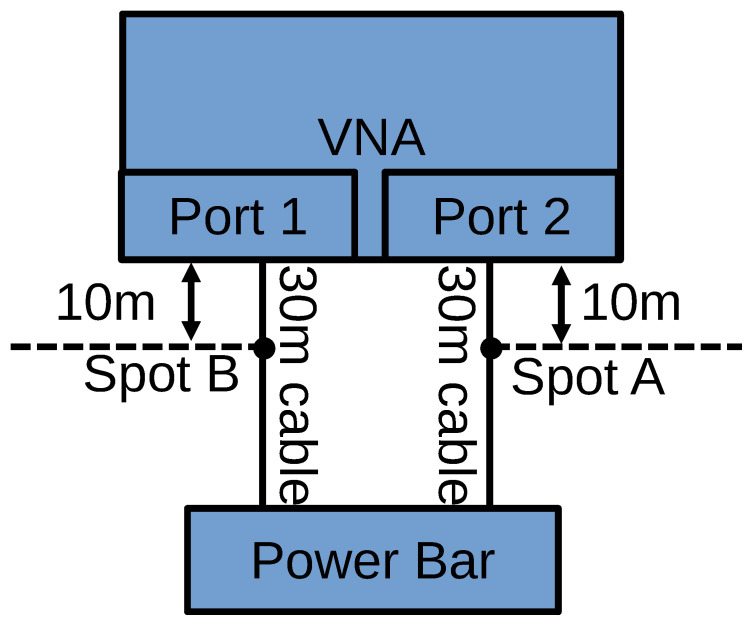
Schematic illustration of the locations of Spots A and B, at which degradations are applied.

The cable connected between VNA Port 1 and the power bar is kept intact during these seven stages. The degradations are introduced incrementally to the cable connected between VNA Port 2 and the power bar in two locations. The first location, referred to as Spot A, is 10 m away from VNA Port 2, while the second location, referred to as Spot B, is 20 m away from VNA Port 2, as shown in Figure 5.

**Figure 6:**
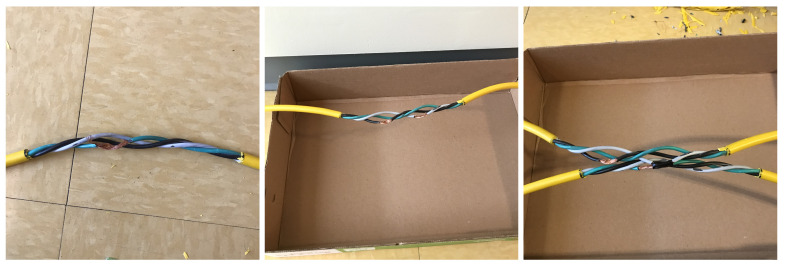
Photos of applied degradations (**Left**) at Spot B after execution of Stage 3, (**Middle**) at Spot A after execution of Stage 5, (**Right**) after execution of Stage 7.

In Stage 1, 20 cm of outer insulation is removed at Spot B. In Stage 2, an additional 20 cm of outer insulation is removed at Spot A. In Stage 3, another 3 cm of insulation and 11 strands of the ground conductor are removed at Spot B. This is shown in Figure 6 (left). In Stage 4, an additional 2 cm of insulation and 9 strands of the neutral conductor are removed at Spot A. In Stage 5, an additional 2 cm of insulation and 7 strands of the live conductor are removed at Spot A, as shown in Figure 6 (middle). In Stage 6, the exposed parts of the neutral conductor are taped to the floor surface. In Stage 7, we revert to the wire-ground taping introduced in Stage 6 and instead short the exposed part of the ground conductor at Spot B with the exposed part of the neutral conductor at Spot A. This is shown in Figure 6 (right).

## 3. PCA-Based Anomaly Detection

In this section, we derive the proposed ML-aided cable monitoring technique. It relies on PCA for lower-dimensional signal representation in conjunction with thresholding of the representation error for anomaly detection. We first introduce our use of clustering as a data pre-processing procedure in Section 3.1. Then, we briefly discuss the principles of PCA in Section 3.2, followed by the description of our application of PCA for anomaly detection in Section 3.3. Details for the implementation of the PCA-based anomaly detection with a focus on the generation of training and testing data are provided in Section 3.4.

### 3.1. Clustering for Data Pre-Processing

In the literature, several clustering techniques have been used as data pre-processing procedures for anomaly detection solutions [39,40,41]. In the context of cable diagnostics, ref. [37] uses density-based spatial clustering of applications with noise (DBSCAN). Typically, dimension reduction techniques are applied to the data before clustering. For this purpose, ref. [37] applies t-distributed stochastic neighbor embedding (t-SNE). In this work, we adapt DBSCAN for clustering and investigate the use of t-SNE, PCA (this use of PCA is different from the subsequent use of PCA for anomaly detection), and independent component analysis (ICA) for dimension reduction before clustering. When conducting PCA-based anomaly detection, we use the *k* nearest neighbour (kNN) classification method to determine the cluster to which a test sample belongs.

### 3.2. PCA Background

PCA is an unsupervised dimension reduction technique [42]. Different from supervised learning, which requires data with labels and close to balanced samples for different classes, the unsupervised learning schemes can process samples from a single class. This fits our case of cable monitoring well as we can use measurements for only healthy cables for training. That is, we do not require measurements of faulty electric components, which would likely be difficult to obtain in a practical scenario.

PCA projects a multi-dimensional data with dimension *d* into a sub-space spanned by *m* principal axes. Suppose we have $n_{\mathrm{Tr}}$ training samples ${\vec{x}}_{i}$, $1\leq i\leq n_{\mathrm{Tr}}$, of dimension *d*. Then, a set of principal axes ${\vec{v}}_{j}$, 1≤j≤m, is learned by the PCA, and each piece of training data ${\vec{x}}_{i}$ is approximated, or reconstructed, as

(4) $${\hat{\vec{x}}}_{i}=\sum_{j=1}^{m}\alpha_{ij}{\vec{v}}_{j},$$

where $\alpha_{ij}\in R$. That is, in the new space spanned by the learned *m* principal axes, each data sample ${\vec{x}}_{i}$ is represented using the *m* coefficients $\alpha_{ij}$, 1≤j≤m. Thus, we achieve a dimensionality reduction from *d* to *m*, where *m* is smaller than *d*. Given the following difference,

(5) $${\vec{\delta }}_{i}={\hat{\vec{x}}}_{i}-{\vec{x}}_{i}=\sum_{j=1}^{m}\left[\alpha_{ij}{\vec{v}}_{j}\right]-{\vec{x}}_{i},$$

the normalized reconstruction error for the training data can be computed as

(6) $$E_{\mathrm{Tr}}=\sqrt{\sum_{i=1}^{n_{\mathrm{Tr}}}{∥{\vec{\delta }}_{i}∥}_{2}^{2}}/\sqrt{\sum_{i=1}^{n_{\mathrm{Tr}}}{∥{\vec{x}}_{i}∥}_{2}^{2}}.$$

The PCA is carried out in such a manner that for each number of principal axes *m*, the normalized reconstruction error for the training data $E_{\mathrm{Tr}}$ is minimized. Moreover, the principal axes are orthonormal. Hence, $\alpha_{ij}={\vec{x}}_{i}{\vec{v}}_{j}^{T}$.

After the PCA is trained, we can apply the PCA to a set of $n_{\mathrm{Ts}}$ test data samples ${\vec{y}}_{i}$, $1\leq i\leq n_{\mathrm{Ts}}$. Then, each test sample ${\vec{y}}_{i}$ is approximated as

(7) $${\hat{\vec{y}}}_{i}=\sum_{j=1}^{m}{\vec{y}}_{i}{\vec{v}}_{j}^{T}{\vec{v}}_{j},$$

and the normalized reconstruction error $E_{\mathrm{Ts}}$ for the test data is computed analogously to (5) and (6).

### 3.3. PCA for Anomaly Detection

One application of PCA is anomaly detection [43], which is also the application considered in this paper. Generally speaking, we use the data gathered during the normal operation of the considered system to train the PCA, and then apply it to obtain a lower-dimensional reconstruction of the test data. If the cable or network under monitoring is healthy, then we expect the reconstruction for the $E_{\mathrm{Ts}}$ test data to be similar to $E_{\mathrm{Tr}}$ for the training data. On the other hand, if the difference $E_{\mathrm{Ts}}-E_{\mathrm{Tr}}$ exceeds a threshold, we consider the behavior anomalous and an alarm is raised.

The choice of the threshold value determines the balance between the DA, i.e., true positive rate, and the FA, i.e., false positive rate. For a higher threshold value Γ, we obtain a higher DA accompanied with a higher FA. This means that the detector is more sensitive to varieties of the potential anomalies but leads to more false alarms when the system is operating under normal conditions. To determine threshold value Γ, we obtain the mean of normalized reconstruction error $E_{\mathrm{Tr}}$ for the training data as μ and standard deviation of the normalized reconstruction error $E_{\mathrm{Tr}}$ for the training data as σ. Then, we obtain certain threshold Γ(λ) as

(8) Γ(λ)=μ+λ·σ.

When we alter the value of λ, we obtain different levels of threshold Γ(λ). For a higher value of λ, we obtain a higher value of Γ(λ).

For a given Γ(λ), the DA is generally dependent on the properties of the faulty test data. For example, for different types and severities of the fault, the same Γ(λ) results in different DAs. However, the properties of the healthy test data should be similar to the properties of the healthy training data used to train the PCA. In that case, we can have an estimate for the FA for the healthy test data given a particular value of λ. For this purpose, we can approximate the distribution of the normalized reconstruction error $E_{\mathrm{Tr}}$ for the training data as the Gaussian distribution. Then, the estimated FA is

(9) $$P_{\mathrm{FA}}=\frac{1}{2}\left[1-\mathrm{erf}\left(\frac{\lambda }{\sqrt{2}}\right)\right],$$

where erf(·) is the Gaussian error function.

For the design of threshold value Γ(λ), we can consider how much FA we can tolerate for the designed system. Then, we substitute that value as $P_{\mathrm{FA}}$ in Equation (9) and solve for the corresponding λ to obtain the corresponding threshold Γ(λ).

### 3.4. Implementation Details

(1) Training: For the generation of training data for the ML model, we take measurements of the overall PLC network in the laboratory setup with cables and loads connected to the power bar as shown in Figure 3 and Figure 4. For the eight different load conditions mentioned in Section 2.3 (see Table 1) with or without energization, we obtain 16 different measurements and thus 16 different two-port networks. For each of the different two-port networks, we randomly generate its terminal impedance load $Z_{L}$. After obtaining all the training samples, we divide these samples into the non-energized group and the energized group. For each group, we conduct the data pre-processing procedure as described in Section 3.1 in an attempt to identify meaningful clusters. We note that if no meaningful clusters are identified, the whole group of data is regarded as a single data cluster. Then, for each identified data cluster, we use training samples from that cluster to train a separate PCA, with its own sets of $E_{\mathrm{Tr}}$ and thus its own values of μ, σ for $E_{\mathrm{Tr}}$.

(2) Testing: For the generation of test data for the ML model, we perform the same measurements as described above but also with the manually introduced degradations as described in Section 2.4. Therefore, we have 16 different two-port network measurements for each of the eight health conditions of the cable. For each of those, we again randomly generate terminal impedance load $Z_{L}$. Then, as described in Section 3.1, we use the kNN algorithm to identify the cluster for each test sample and apply the PCA anomaly detector trained for the corresponding data cluster.

(3) CFR Generation: Denoting the ABCD matrix of the two-port network measurement as

(10) $$M_{\mathrm{meas}}=\left[\begin{array}{cc} A_{\mathrm{meas}} & B_{\mathrm{meas}}\\ C_{\mathrm{meas}} & D_{\mathrm{meas}} \end{array}\right],$$

we obtain the channel frequency response of the corresponding two-port network terminated with $Z_{L}$ as

(11) $$H_{\mathrm{meas}}=Z_{L}/\left(A_{\mathrm{meas}}Z_{L}+B_{\mathrm{meas}}\right).$$

We note that $H_{\mathrm{meas}}$ is a vector of complex values for different frequencies. As in previous work [8,33,44] and similar to using the SNR in [13,36,37], we use normalized vectors of magnitudes as training and test samples. For both training and test samples, the mean and the standard deviation are used for normalization.

## 4. Results

In this section, we present the measurement and numerical results for the considered system setup and anomaly detection method. The measurements are taken in the frequency range from 1.8 MHz to 30 MHz. We sample the computed frequency responses $H_{\mathrm{syn}}$ and $H_{\mathrm{meas}}$ in intervals of 24.4 kHz to mimic the frequency responses estimated with commercially available PLC systems using orthogonal frequency division multiplexing.

### 4.1. Calibration and Measurement of Components

**Figure 7:**
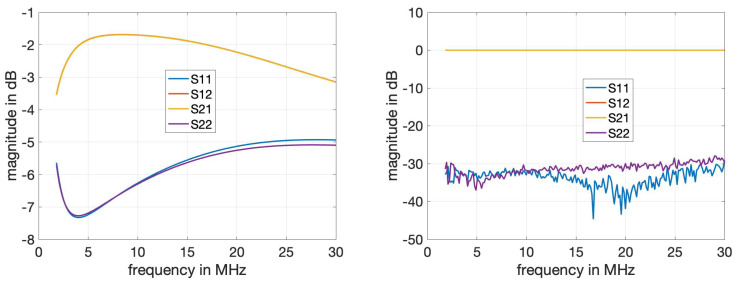
Magnitude of measured S-parameters for the series connection of the two PLC couplers before (**left**) and after calibration (**right**). The curves for the $S_{12}$ and $S_{21}$ parameters completely overlap.

Figure 7 shows the S-parameter measurements for a series connection of the two PLC couplers. We observe that before calibration, transmission parameters $S_{12}$ and $S_{21}$ experience attenuation, and reflection coefficients $S_{11}$ and $S_{22}$ are fairly significant. After calibration, the transmission coefficients have the desired 0 dB magnitude and the reflection coefficients are well suppressed at about −30 dB for the considered frequency range. This shows that the calibration is effective and the results for the measurement of the DUT are not affected by the ancillary components.

**Figure 8:**
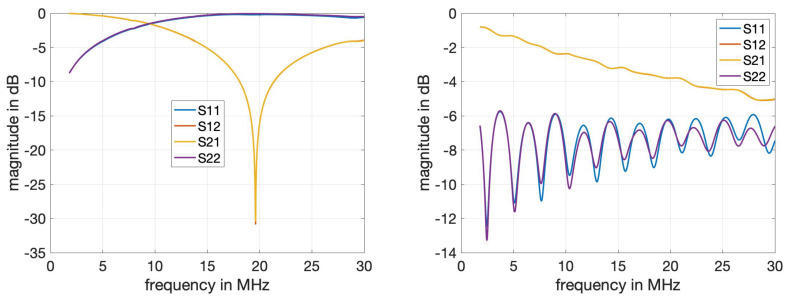
Measured S-parameters for the power bar (**left**) and the 30 m power cable (**right**) as the DUT. The curves for the $S_{12}$ and $S_{21}$ parameters completely overlap. The $S_{11}$ and $S_{22}$ parameters for the power bar (**left**) match very closely.

Figure 8 shows the S-parameter measurements after calibration with the power bar and the 30 m cable as the DUT, respectively. We observe that the signal transmitted through the power bar experiences a strong attenuation from 15 MHz to 25 MHz. As this renders de-embedding via calibration difficult and measurement results less reliable within the 15–25 MHz range, we focus on the frequency band from 5 to 15 MHz for the results presented in later sections. For the characteristics of cables, we note a stronger attenuation for increasing frequencies as expected, while the reflections indicate the impedance mismatch at the input and output ports.

### 4.2. Data Generation and Pre-Processing

As mentioned above, we use the frequency band from 5 MHz to 15 MHz, which corresponds to 410 frequency points. We generate the terminal load impedance $Z_{L}$∼(U[0,50]+jU[−50,50]) Ω as in [8], where U[a,b] denotes the uniform distribution between *a* and *b*. We synthetically generate $n_{\mathrm{Tr}}$ = 20,000 samples for training by varying impedances at terminal loads and by randomly choosing one of the eight different load conditions in the power bar for the measurement, as shown in Table 1. For each of the eight stages of the manually introduced degradation, we synthetically generate $n_{\mathrm{Ts}}=5000$ frequency response samples by varying impedances at terminal loads and by randomly choosing one of the eight different load conditions in the power bar for the measurement. Since we have eight stages of degradations, including the intact cable state as Stage 0, we have a total of 40,000 test samples.

**Figure 9:**
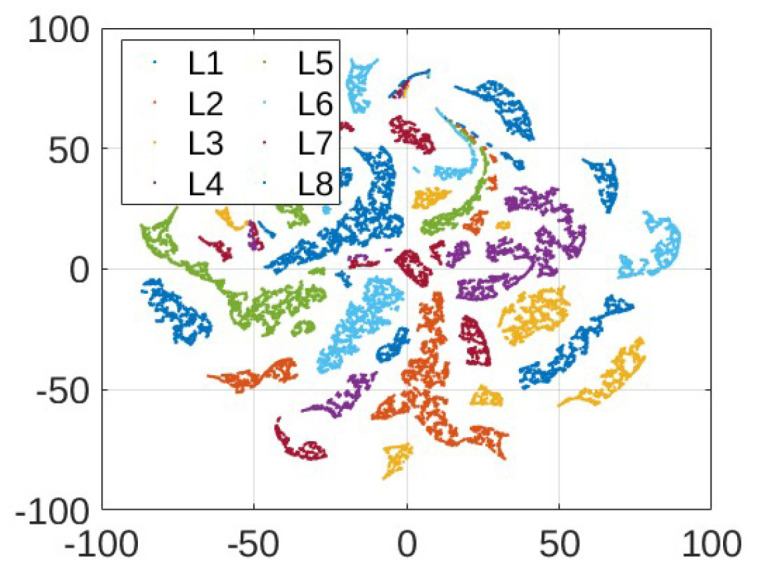
Visualization of two-dimensional t-SNE transformed energized training data.

**Figure 10:**
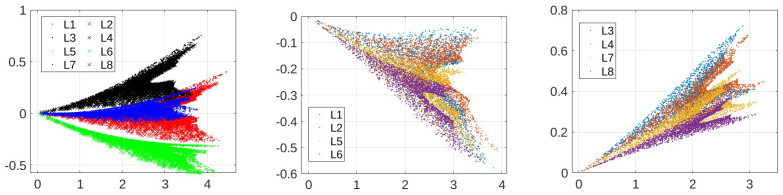
Visualization of embedded data after dimension reduction using ICA for non-energized (**left**) and energized training data (**middle**) and (**right**).

**Figure 11:**
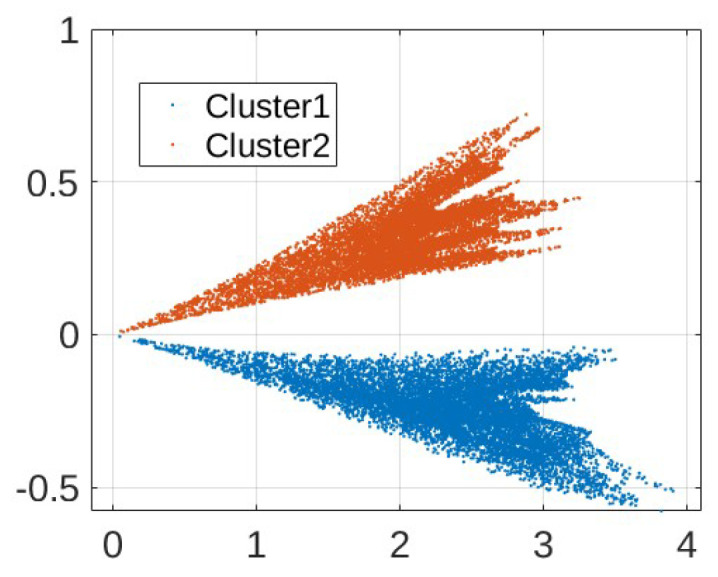
Clustering results using DBSCAN after ICA for energized training samples.

After we obtain samples for training and testing, we apply clustering as a data-preprocessing procedure. Clustering operates on dimension-reduced data. Figure 9 shows the results (we note that the quantities are projections of unitless CFR magnitudes and thus also unitless) when the 410-dimensional CFR training data are reduced to two dimensions using t-SNE, where labels L1-L8 correspond to the eight different load conditions as specified in Table 1. While we observe a separation of two-dimensional data points, a meaningful clustering structure is not immediately clear. Figure 10 shows the results for ICA. A structure that seems to lend itself better to clustering is obtained. In particular, two clusters are identified by DBSCAN for the energized case as shown in Figure 11. On the other hand, it remains difficult to pick out meaningful data clusters for the non-energized case. Hence, we treat the training samples for the non-energized scenario as a single data cluster. Very similar results were obtained when using PCA for dimension reduction prior to the clustering. For the following result, we use ICA and reduction to two dimensions, and kNN with k=5 is used to determine the cluster during testing.

### 4.3. PCA Results

After clustering as a data pre-processing procedure, we apply the PCA-based anomaly detector for each of the identified clusters. Anomaly detection is performed with PCA and m=10 dimensions. To illustrate the benefits of clustering, we show the results with and without clustering and for different thresholds Γ(λ) from (8) in Table 2a,b. Since non-energized case clustering did not result in a partitioning, the results are the same as for those without clustering and only reported in Table 2b.

Comparing the results in Table 2a,b, we observe significant performance improvements due to clustering. We note that clustering partitions the training data set into only two clusters as shown in Figure 11, despite the eight load conditions as shown in Figure 10. However, this clustering is effective, as the load changes within the same cluster, e.g., within L1, L2, L5, L6, do not cause substantial changes to CFR measurements (see discussion below).

Next, concentrating on the absolute performance of anomaly detection, we observe from the results in Table 2a that we achieve near-perfect DA for all the considered thresholds and for all stages of manually introduced degradations. This suggests that our proposed cable anomaly detection solution is able to reliably detect potential cable anomalies with high DA and low FA. In particular, we expect that online diagnostics would be applied to cables operating in an energized state. For the non-energized setup, the results shown in Table 2b display a trade-off between DA and FA. In particular, with a higher value of λ, we enforce a lower FA but at the price of an also reduced DA. Furthermore, as can be expected, we observe better results for more severe faults.

### 4.4. Discussion

**Figure 12:**
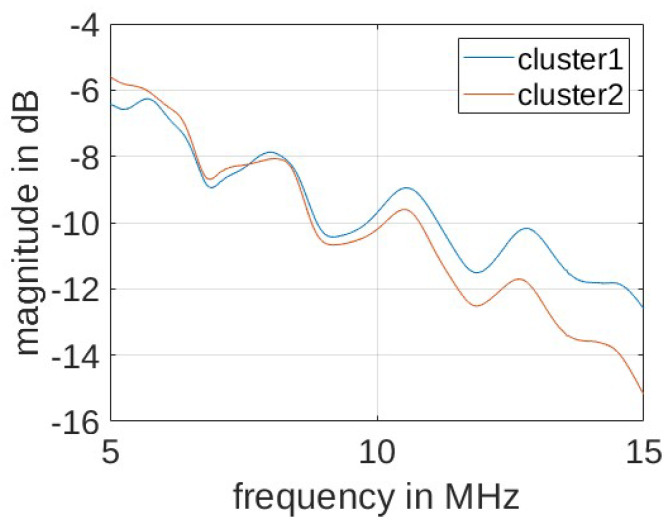
Average CFR magnitude across cluster samples.

The comparison between the results shown in Table 2a,b suggests that the identification of meaningful data clusters is significant for the performance of the proposed PCA-based anomaly detection solution. As shown in Figure 11, the data for the energized scenario form two clusters vary because of the various load conditions. In particular, the data collected with the electric fan unplugged corresponding to load conditions L1, L2, L5, and L6 in Figure 10 (middle) are aggregated as Cluster 1, while the data collected with the operating fan corresponding to load conditions L3, L4, L7, and L8 in Figure 10 (right) are aggregated as Cluster 2. This is in agreement with our measurement experience, where in the energized scenario, the electric fan has the largest impact on the S-parameter measurement result when compared with the other considered loads. Figure 12 illustrates this in terms of the average CFR magnitude across samples from the two clusters as shown in Figure 12. The application of the ICA makes this difference more evident so that clustering becomes effective. This has important implications for the practical scenario, where the load impedances of the grid are constantly changing (We note that different load impedances could also have an influence through affecting line parameters due to resulting current and temperature fluctuations). In such occasions, the clustering algorithm is able to explore the internal structure of the collected data and identify several typical load conditions corresponding to different data clusters.

Even for the non-energized setup, for which the considered clustering algorithm is not able to identify meaningful clusters, we observe the presence of an internal data structure as shown in the left subfigure of Figure 10. If information about the load condition is available during training, then one can partition training samples accordingly and train anomaly detectors for each of the load conditions. Applying this procedure to our data set led to perfect DA for any degradation stage and for all considered thresholds Γ(λ) used in Table 2a for both the non-energized and the energized scenario. This emphasizes the role of pre-processing to differentiate load-induced, i.e., normal variations from anomalies.

## 5. Conclusions

In this paper, we use laboratory measurements of scattering parameters of a power line communication network to validate a cable anomaly detection scheme. The technical innovation of this work lies in the proposed method for anomaly detection, which uses channel frequency response measurements as input and consists of clustering as a data pre-processing step followed by anomaly detection using the representation accuracy of principal component analysis. The conceptual innovation lies in the way experimental measurement data are augmented and used for validation. This procedure could also be applied to confirm other diagnostic schemes using power line signals. The overall anomaly detection model is trained in an unsupervised fashion, which allows us circumvention of the requirement of costly-to-obtain faulty measurements typical for conventional supervised schemes. Our measurements include different load conditions and energized and non-energized setups to mimic a practical scenario. Furthermore, measurements with manually introduced degradations to the cables are obtained for the numerical evaluation of our proposed scheme. The performance results obtained with the developed scheme support the hypothesis that reliable cable monitoring using PLC channel frequency response data is possible. For future work, it will be meaningful to extend measurements with actual low- or medium-voltage power cables and loads deployed in such power grids so as to confirm or adjust conclusions drawn in this work. Furthermore, comparisons of different cable monitoring schemes and possible variations of them using such data will be an important step towards their practical deployment.

## Figures and Tables

**Table 1:** Enumeration of load conditions using the three different loads.

Load Condition	Kettle	Fan	Monitor
L1	-	-	-
L2	✓	-	-
L3	-	✓	-
L4	✓	✓	-
L5	-	-	✓
L6	✓	-	✓
L7	-	✓	✓
L8	✓	✓	✓

**Table 2:** Detection accuracy (DA) and false alarm rate (FA) results for energized and non-energized setups. Threshold Γ(λ) for λ=1,2,3. (**a**) DA and FA for energized setup for the different degradation stages with the application of clustering. Results for non-energized case are as in Table 2b. (**b**) DA and FA for non-energized setup (**top**) and energized setup (**bottom**) for the different degradation stages and without the application of clustering.

(**a**)				
	**Threshold**	Γ(1)	Γ(2)	Γ(3)
	Stage 0 FA	13%	5.4%	2.3%
	Stage 1 DA	1	1	1
	Stage 2 DA	1	1	1
	Stage 3 DA	1	1	1
	Stage 4 DA	1	1	1
	Stage 5 DA	1	1	1
	Stage 6 DA	1	1	1
	Stage 7 DA	1	1	1
(**b**)				
	**Threshold**	Γ(1)	Γ(2)	Γ(3)
non-energized	Stage 0 FA	14%	5.4%	1.9%
	Stage 1 DA	48%	16%	5.3%
	Stage 2 DA	51%	20%	6.2%
	Stage 3 DA	57%	30%	13%
	Stage 4 DA	70%	44%	24%
	Stage 5 DA	77%	52%	33%
	Stage 6 DA	76%	51%	31%
	Stage 7 DA	1	99%	97%
energized	Stage 0 FA	15%	5.2%	1.7%
	Stage 1 DA	44%	19%	7.6%
	Stage 2 DA	44%	19%	7.9%
	Stage 3 DA	75%	39%	20%
	Stage 4 DA	93%	63%	35%
	Stage 5 DA	99%	78%	43%
	Stage 6 DA	99%	75%	42%
	Stage 7 DA	1	99%	96%

## Data Availability

The data used and methods presented in this study are openly available at https://github.com/ubcyinjia/ValidationCableMonitoring.
